# Protein-Enabled Size-Selective Defect-Sealing of Atomically Thin 2D Membranes for Dialysis and Nanoscale Separations

**DOI:** 10.1021/acs.nanolett.4c04706

**Published:** 2024-12-23

**Authors:** Peifu Cheng, Nicholas Ferrell, Saban M. Hus, Nicole K. Moehring, Matthew J. Coupin, Jamie Warner, An-Ping Li, William H. Fissell, Piran R. Kidambi

**Affiliations:** † Department of Chemical and Biomolecular Engineering, Vanderbilt University, Nashville, Tennessee 37212, United States; ‡ Division of Nephrology, Department of Internal Medicine, The Ohio State University Wexner Medical Center, Columbus, Ohio 43210, United States; ◊ Center for Nanophase Materials Sciences, Oak Ridge National Laboratory, Oak Ridge, Tennessee 37831, United States; ∥ Interdisciplinary Materials Science Program, Vanderbilt University, Nashville, Tennessee 37212, United States; ⊥ Department of Medicine and Division of Nephrology and Hypertension, Vanderbilt University Medical Center, Nashville, Tennessee 37232, United States; Δ Vanderbilt Institute of Nanoscale Sciences and Engineering, Vanderbilt University, Nashville, Tennessee 37212, United States; § Walker Department of Mechanical Engineering, University of Texas at Austin, Austin, Texas 78712-1591, United States; 8 Department of Mechanical Engineering, Vanderbilt University, Nashville, Tennessee 37212, United States

**Keywords:** atomically thin membranes, graphene, protein-enabled defect sealing, nanoscale separations, highly selective membranes

## Abstract

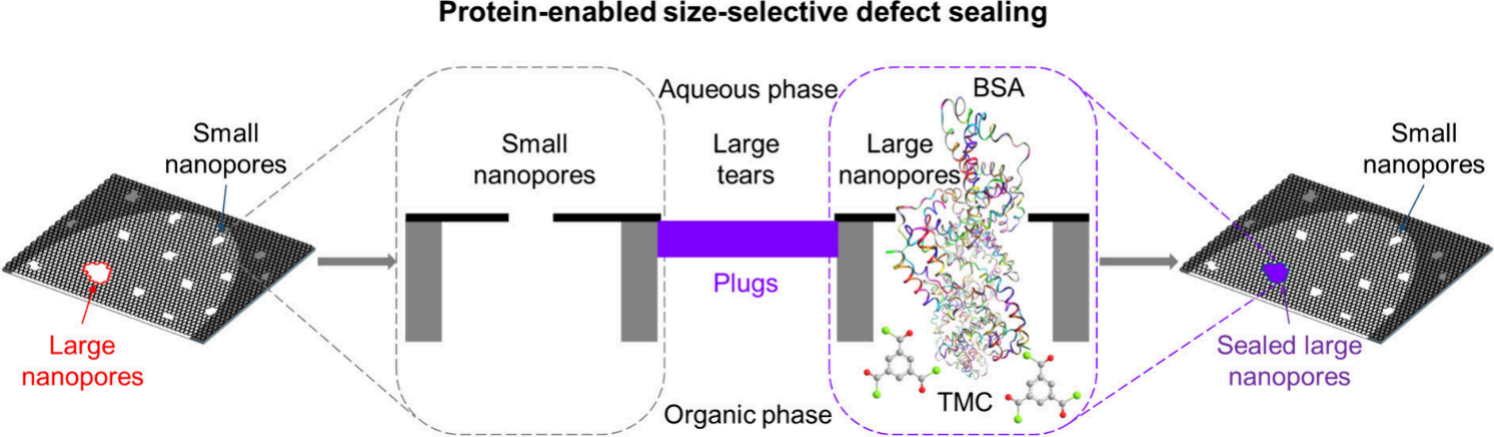

Atomically thin 2D materials present the potential for advancing membrane separations via a combination of high selectivity (from molecular sieving) and high permeance (due to atomic thinness). However, the creation of a high density of precise nanopores (narrow-size-distribution) over large areas in 2D materials remains challenging, and nonselective leakage from nanopore heterogeneity adversely impacts performance. Here, we demonstrate protein-enabled size-selective defect sealing (PDS) for atomically thin graphene membranes over centimeter scale areas by leveraging the size and reactivity of permeating proteins to preferentially seal larger nanopores (≥4 nm) while preserving a significant amount of smaller nanopores (via steric hindrance). Our defect-sealed nanoporous atomically thin membranes (NATMs) show stability up to ∼35 days during size-selective diffusive separations with a model dialysis biomolecule fluorescein isothiocyanate (FITC)-Ficoll 70 in phosphate buffer saline (PBS) solution as well as outperform state-of-the-art commercially available dialysis membranes (molecular-weight-cutoff ∼3.5–5 kDa and ∼8–10 kDa) with significantly higher permeance for smaller solutes KCl (∼0.66 nm) ∼5.1–6 × 10^–5^ ms^–1^ and vitamin B12 (B12, ∼1.5 nm) ∼2.8–4 × 10^–6^ ms^–1^ compared to small protein lysozyme (Lz, ∼4 nm) ∼4–6.4 × 10^–8^ m s^–1^, thereby allowing unprecedented selectivity for B12/Lz ∼70 and KCl/Lz ∼1280. Our work introduces proteins as nanoscale tools for size-selective defect sealing in atomically thin membranes to overcome persistent issues and advance separations for dialysis, protein desalting, small molecule separations/purification, and other bioprocesses.

Membrane separations have enabled transformative advances in desalination,^1−5^ nanofiltration,^3−9^ ionic/molecular separation,^4,7,10−17^ proton transport,^18−23^ energy harvesting,^24,25^ gas separation,^26−28^ and dialysis,^10,25,30,31^ among other fields.^32−29^ Most conventional membranes comprise a selective polymer layer embedded with nanoscale pores. Diffusion in such polymeric pores is increasingly hindered as the molecule size approaches the pore dimension.^37^ Hence, highly permeable polymers typically provide less selectivity, while making the polymer denser to achieve higher selectivity comes at the expense of permeance.

Nanoporous atomically thin membranes (NATMs) based on two-dimensional (2D) materials present the potential to overcome these persistent challenges in membrane separations. The atomic thinness,^24,25^ the ability to sustain a high-density of nanopores^3−11,16,35^ and the tunability of pore size distributions^3−11,38^ present potential to realize high selectivity via molecular sieving while maintaining high permeance. Top-down approaches like ion bombardment/irradiation,^16,35^ chemical oxidation,^6^ UV/ozone etching^4,7,39^ and plasma treatment^3,8−10,38^ have been used in conjunction with graphene synthesized via scalable chemical vapor deposition (CVD) processes^40−43^ to introduce high-density nanopores. Top-down processes usually form a heterogeneous pore size distribution with an undesired long tail,^4,5,10,44^ since pre-existing/intrinsic CVD graphene defects exhibit a much higher propensity to enlarge than nucleation of new defects in the pristine lattice.^45^ A few large defects can lead to disproportionately high nonselective leakage compromising 2D membrane performance.

On the other hand, precisely controlling the nanopore size in scalable NATMs via bottom-up approaches remains extremely challenging,^11,25,46,47^ and a few larger pores or tears are unavoidable during fabrication^48−51^ but can severely compromise the membrane performance. Hence, nanopore heterogeneity represents the Achilles’ heel of NATMs and developing novel methods to enable a narrow nanopore size distribution is imperative to advance NATMs.

Prior studies have demonstrated the mitigation of nonselective leakage by leveraging charge based interactions or chemical reactions,^4−8,10,38^ as well as independent stacking of multilayer nanosheets.^20,52,53^ However, achieving sharp nanoscale separation of ions/molecules within close size ranges for dialysis, desalting, and other bioprocesses remains challenging and size-selective defect-sealing of large defects in scalable NATMs remains nontrivial. This is further exacerbated by conformational changes as well as physical deformation that biomolecules undergo during transport through nanopores.^37,54,55^ Hence, achieving membranes that allow for size-selective biomolecule separation has remained elusive, with conventional membranes primarily comprising polydisperse, rough, tortuous pores embedded in a polymer with hindered diffusion.

To overcome these persistent issues, here, we fabricate highly selective graphene NATMs via a facile and novel protein-enabled defect sealing (PDS) approach. We select bovine serum albumin (BSA) as the protein molecule to react with trimesoyl chloride (TMC) by introducing them on opposite sides of graphene to seal larger nanopores (≥4 nm) while successfully preserving a significant amount of smaller nanopores (<4 nm). Our rationale for selecting BSA as the protein is to demonstrate NATM applications such as dialysis that allow for small (<4 nm) molecule permeation while mitigating transport of BSA. To the best of our knowledge such an approach has not been demonstrated with proteins and our successful demonstration unravels the wide library of biomolecules (proteins, DNA, RNA, etc.) as precise nanoscale tools for NATM fabrication.

Compared with state-of-the-art commercially available dialysis membranes (molecular-weight-cutoff ∼3.5–5 kDa and ∼8–10 kDa) and laboratory-scale graphene membranes, the fabricated graphene NATMs offer significant advances with higher permeance of smaller solutes (∼5.1 × 10^–5^–6 × 10^–5^ m s^–1^ for KCl and ∼2.8 × 10^–6^–4 × 10^–6^ m s^–1^ for vitamin B12) and negligible permeance of larger molecules (∼4 × 10^–8^–6.4 × 10^–8^ m s^–1^ for lysozyme) along with ultrahigh selectivity (up to ∼70 for vitamin B12/lysozyme and up to ∼1280 for KCl/lysozyme). We further evaluate the diffusive performance of graphene NATMs with model molecules and show they surpass conventional dialysis membranes (regenerated cellulose and polyacrylonitrile) with high permeability (up to 2 orders of magnitude higher) for small and middle molecules as well as very limited permeability for large molecules (MW > 50 kDa).

To fabricate centimeter-scale atomically thin membranes, monolayer CVD graphene was initially transferred onto polycarbonate track etched (PCTE) supports via isopropanol-assisted hot lamination (IHL, see Methods section).^4,30^ Subsequently, an O_2_ plasma (OP) etch was used to introduce a high density of defects (that function as nanopores in the 2D lattice of graphene) in the atomically thin membrane.^8−10,56^ Finally, size-selective protein-enabled defect sealing (PDS) with bovine serum albumin (BSA) as a monomer (see Methods) was used to seal large nanopores and tears (Figure 1A) while retaining smaller pores.

**Figure 1:**
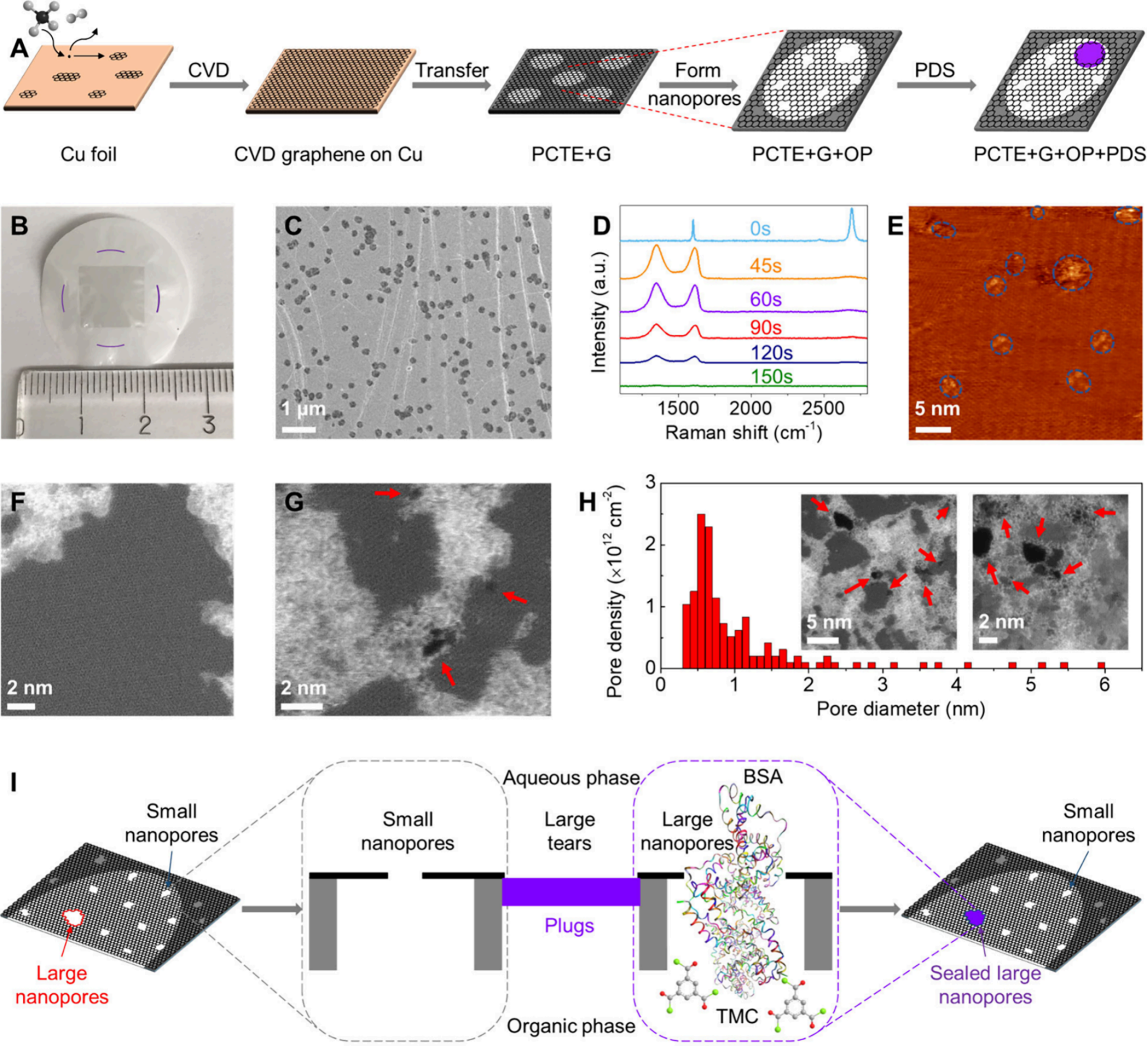
Fabrication and characterization of large-area nanoporous atomically thin membranes (NATMs) with protein-enabled size-selective defect sealing. A) Schematic of the fabrication process of graphene NATMs. Monolayer graphene (G) synthesized via CVD on Cu foil was first transferred onto PCTE support via isopropanol-assisted hot lamination (IHL, see Methods section). After the Cu foil was etched away, the transferred graphene on PCTE support (PCTE+G) was treated by O_2_ plasma (OP) to introduce new defects as well as enlarge existing intrinsic defects in the graphene lattice (PCTE+G+OP). Finally, protein-enabled size-selective defect sealing with BSA in the PBS solution (aqueous phase) and TMC in hexane (organic phase) was used to seal large nanopores and tears while preserving preferred small nanopores in the graphene membrane (PCTE+G+OP+PDS). B) Optical image of graphene NATM. The black square shows the graphene area, while the purple dashed circle represents the membrane region subjected to the PDS process. C) SEM image of pristine graphene transferred onto the PCTE support. The dark circles indicate PCTE cylindrical pores (∼200 nm diameter) covered by graphene. D) Raman spectra of CVD graphene (transferred onto 300 nm SiO_2_/Si wafer) exposed to the plasma of O_2_ for different times. E) STM image of graphene on Cu foil after O_2_ plasma treatment for 90 s. Blue circles indicate nanopores and vacancy defects in the lattice. F) Atomic resolution STEM image of as-synthesized CVD graphene. G) STEM image of graphene after O_2_ plasma treatment for 90 s. Also see the insets in H). Red arrows indicate defects or nanopores in the graphene lattice. H) Measured pore size distribution from STEM images of O_2_ plasma (90 s) treated graphene lattice. Also see the calculated pore size distribution in Supporting Information, Figure S2 when considering both the carbon electron diameter (∼0.13 nm) and carbon van der Waals (VDW) diameter ∼0.34 nm. I) Schematic of O_2_ plasma treated graphene (with small and large nanopores) suspended over single PCTE pores, subjected to the PDS process to selectively seal large nanopores and tears. Sealing plugs are expected to mainly form when BSA molecules (with short axis ∼4 nm diameter^65^) diffuse through the large nanopores (≥4 nm) in graphene lattice into the organic phase and react with TMC.

The optical image of the synthesized graphene NATM (Figure 1B) shows centimeter scale nanoporous graphene (dark square) on PCTE supports. The graphene region subjected to size-selective PDS can be identified by the circular Franz cell edge (purple dashed line). Scanning electron microscopy (SEM) images indicate successful large-area transfer of graphene onto the PCTE support (Figure 1C, darker 200 nm circles) with coverage >99%, as indicated by normalized ethanol leakage ∼0.7% (PCTE+G/PCTE, Figure 2A). Raman spectra (Figure 1D) confirm the high quality of as-synthesized monolayer graphene (2D ∼2690 cm^–1^, G ∼1600 cm^–1^ and *I*_2D_/*I*_G_ ∼ 1.6) with the absence of D peak ∼1350 cm^–1^ as well as an increase in defects with increasing exposure times to O_2_ plasma treatment.^8−10,56^ Scanning tunneling microscopy (STM) images (Figure 1E) acquired directly on the O_2_ plasma treated graphene on Cu foil (to avoid residues from transfer) further confirms the creation of defects (marked by blue circles) in the graphene lattice.^57,58^ Atomic resolution scanning transmission electron microscopy (STEM) images show the presence of significantly more defects/nanopores in the graphene lattice after the O_2_ plasma (compare Figure 1F with Figure 1G and Figure 1H insets) as well as inevitable residues from transfer despite polymer free transfer processes. The pore size distribution computed from the observable nanopores in STEM images shows most nanopores <1.2 nm, some nanopores are in the range 1.2–4 nm, and a few nanopores >4 nm (Figure 1H) and an overall nanopore density ∼1.4 × 10^13^ cm^–2^ (Figure 1H). Although O_2_ plasma successfully introduces a high density of defects in the graphene lattice, it typically results in a distribution of pore sizes with a few large nanopores in the tail (Figure 1H, also see Figure S2),^5,6,10,34,44^ which could allow for nonselective leakage compromising membrane selectivity.

**Figure 2:**
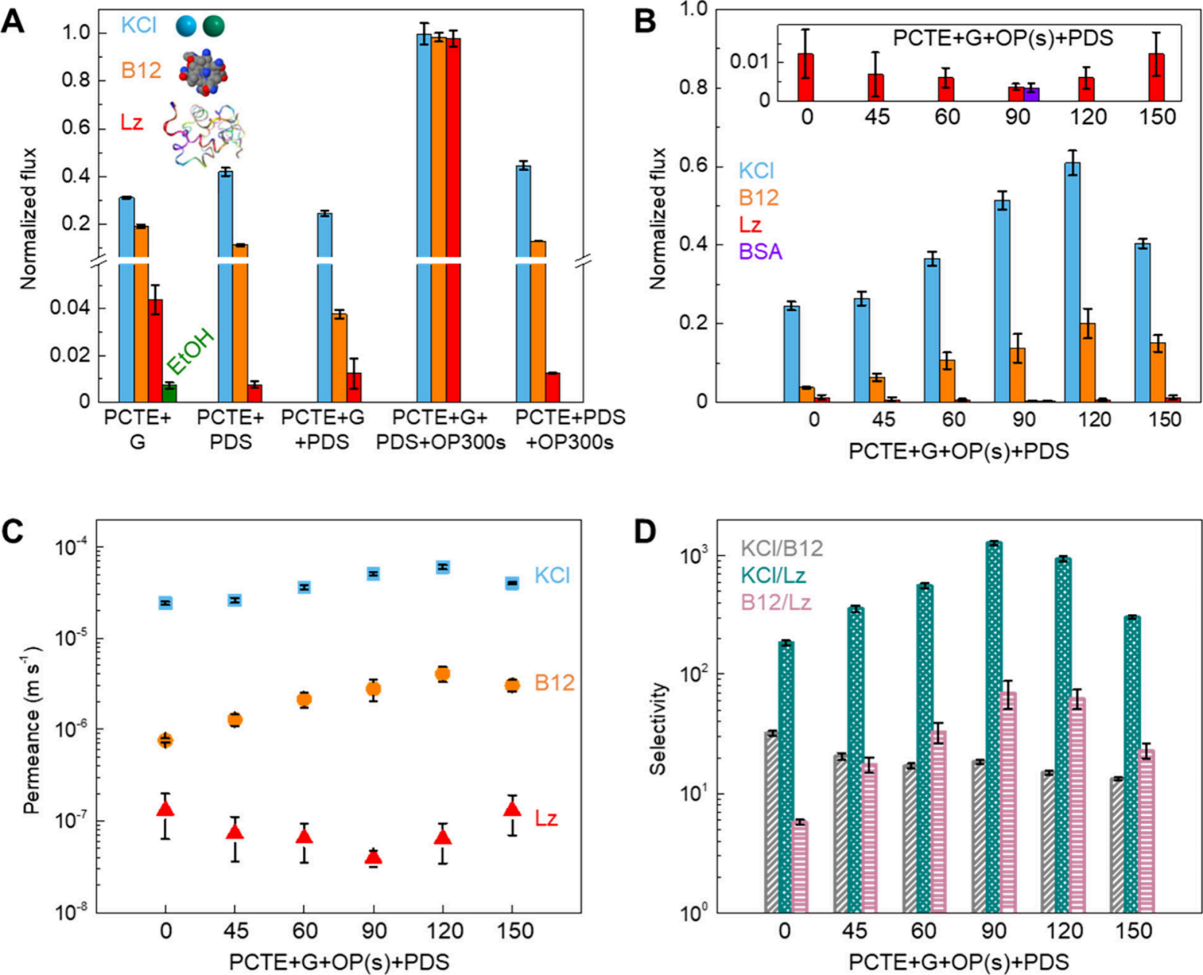
Influence of pore creation (by O_2_ plasma etch) and PDS on the performance of graphene NATMs. A) Diffusive flux normalized with respect to bare PCTE support for different membranes (PCTE+G, PCTE+PDS, PCTE+G+PDS, PCTE+G+PDS+OP300s, and PCTE+PDS+OP300s) measured using model solutes. B) Normalized flux, C) diffusive permeance (accounting for PCTE support porosity of ∼10%) and D) selectivity (ratio of permeance of two species) of fabricated NATMs. Error bars indicate one standard deviation. Also, see Supporting Information, Figure S4.

To seal the large nanopores in O_2_ plasma treated graphene as well as any large tears from processing/handling, while preserving the small nanopores, we leverage an interfacial reaction between amine groups on a protein molecule (bovine serum albumin, BSA, ∼66 kDa) in the aqueous phase and trimesoyl chloride (TMC) in the organic phase (Figure 1I). We ensure BSA remains in its native (N) form by using pH ∼ 7.4 (pH range for N form ∼4.5–8.0), avoiding higher (pH ∼ 8.0–9.0, BSA undergoes a basic N–B transition) or lower (pH ∼ 3.5–4.5, BSA undergoes a transition from the N form to the F form) values.^59−64^ Notably, the BSA molecule has an elongated ellipsoidal shape^65^ with the short axis ∼4 nm, allowing its permeation through nanopores ≥4 nm, while transport through nanopores <4 nm is expected to be sterically hindered allowing for their preservation. Proteins and biomolecules are known to exhibit some level of squishiness and/or deformation when transporting through nanopores;^37^ hence, our rationale for selecting BSA as the protein molecule is it represents the ideal size of prototypical protein molecule to retain in dialysis applications while allowing other smaller molecules to permeate. Since TMC decomposes in water,^4,7,66,67^ BSA has to diffuse into hexane to react with TMC; i.e., BSA has to transport through the large nanopores or tears in the graphene layer (transport through smaller pores is sterically hindered) to react with TMC. Hence, the reaction interface is pinned within the organic phase, i.e., behind graphene and within the PCTE support, preventing an undesirable coating on the graphene surface (Figure 1I).

The performance of the fabricated graphene NATMs was evaluated via diffusive transport (Figure 2) using a customized side-by-side diffusion cell system^4,6,10,11,16,30,56,68,69^ (see Figure S1) with three model solutes covering a range of sizes, i.e., KCl (salt, hydrated diameter of K^+^ ∼0.662 and Cl^–^ ∼0.664 nm, 74.55 Da),^24^ vitamin B12 (B12, vitamin, ∼1−1.5 nm, 1355 Da),^70^ and lysozyme (Lz, protein, ∼3.8–4 nm, 14.3 kDa).^10^ The as-synthesized graphene transferred onto the PCTE support (Figure 2A) shows normalized fluxes of KCl ∼31.2%, B12 ∼19.1% and Lz ∼4.4%, respectively, consistent with the prior literature.^20,30^ After sealing the large defects/tears in graphene membranes (PCTE+G+PDS, Figure 2A) via the interfacial reaction with BSA and TMC, the normalized fluxes decrease, i.e., KCl ∼24.5%, B12 ∼3.8% and Lz ∼1.2% (PCTE+G+OP0+PDS, Figure 2B). We emphasize that transport through graphene NATMs could arise from i) selective transport through small nanopores (<4 nm) in graphene, ii) nonselective leakage through tears and large nanopores (≥4 nm) in graphene, and iii) transport through the plugs (Figures 1 and 2).

To confirm the reduction in normalized flux stems from sealing of large defects/tears and not the formation of layer over graphene, we etched the graphene with 300 s of O_2_ plasma (see Methods section) and remeasured diffusive flux on the same membrane.^30,69^ Etching the graphene (PCTE+G+PDS+OP300s) results in an increase in normalized flux (KCl ∼99.7%, B12 ∼98.3%, and Lz ∼97.9%, Figure 2A) approaching bare PCTE, indicating that the BSA-TMC reaction does not form an undesirable coating over the graphene, and the observed transport properties are indeed from graphene. Notably, PCTE+PDS controls (normalized fluxes of KCl ∼42%, B12 ∼1.3% and Lz ∼0.8%) do not show significant changes with 300 s of O_2_ plasma (PCTE+PDS+OP300s, KCl ∼44.6%, B12 ∼13% and Lz ∼1.3%) indicating the stability of PDS plugs compared to graphene (Figure 2A).

Next, we systematically evaluated the impact of nanopore formation via the O_2_ plasma etch in conjunction with size-selective protein-enabled defect sealing for the NATMs. The normalized flux of KCl after O_2_ plasma etch and defect sealing (PCTE+G+OP (s)+PDS) systematically increases from ∼24.5% to ∼60.9% with increasing O_2_ plasma time from 0 to 120 s and then decreases to ∼40.4% at 150 s (Figure 2B). The normalized flux of B12 also shows a similar trend with an increase from ∼3.8% to ∼20% with an increasing O_2_ plasma time up to 120 s and then a decrease to ∼15% at 150 s (Figure 2B). However, for Lz, the normalized fluxes are very low (mostly <1.2%, see Figure 2B inset), with the lowest value ∼0.4% at 90 s O_2_ plasma. Notably, membranes with an O_2_ plasma treatment for 90 and 120 s (PCTE+G+OP90+PDS and PCTE+G+OP120+PDS) showed the highest transport of KCl (normalized flux ∼51.4%–60.9%) and B12 (normalized flux ∼13.8%–20%) as well as very low transport of Lz (normalized flux ∼0.4%–0.6%). Additionally, we also measured diffusive transport of BSA through the PCTE+G+OP90+PDS membrane (Figure 2B) and obtained normalized flux of BSA ∼0.3%, which is slightly lower than Lz ∼0.4%.

Taken together, the higher normalized fluxes of KCl and B12 compared to Lz for all PCTE+G+OP+PDS membranes demonstrate that our approach allows for the creation of a high density of nanoscale pores via the O_2_ plasma etch (consistent with Figure 1D,E,G,H) and size-selective sealing of large nanopores (≥4 nm) via the BSA-TMC interfacial reaction (Figure 1I). Furthermore, diffusion-driven transport experiments with different-batch graphene NATMs (Figure S3) show remarkably consistent results, indicating the reliability and reproducibility of the entire process including graphene synthesis, transfer, O_2_ plasma treatment, and protein-enabled size-selective defect sealing.

Longer O_2_ plasma etch times (150 s) can lead to enlarging existing pores and/or merging of adjacent pores together, which could lead to a larger fraction of the membrane area being sealed by the BSA-TMC interfacial reaction, resulting in decreased normalized flux of solutes <4 nm (KCl ∼40.4% and B12 ∼15%) while the normalized flux of larger solutes ≥4 nm (Lz ∼1.2%) through the plugs remains relatively similar to the other defect sealed membranes (Figure 2B).

We also compute diffusive permeance (Figure 2C) and selectivity (ratio of permeance of each species, Figure 2D) for the NATMs by accounting for the PCTE support porosity ∼10% (see the Methods section, also see Figure S4 for permeance without accounting for PCTE support porosity). The trends in permeance follow the normalized flux; i.e., the permeance of KCl through PCTE+G+OP+PDS membranes gradually increases from ∼2.4 × 10^–5^ to ∼6 × 10^–5^ m s^–1^ with increasing O_2_ plasma time to 120 s and then decreases to ∼4 × 10^–5^ m s^–1^ at 150 s. Similarly, the permeance of B12 shows a continuous increase from ∼7.6 × 10^–7^ to ∼4 × 10^–6^ m s^–1^ with the increase of O_2_ plasma time from 0 to 120 s and then a moderate decrease to ∼3 × 10^–6^ m s^–1^ at 150 s. In contrast, the permeance of Lz first decreases from 1.3 × 10^–7^ to ∼4 × 10^–8^ m s^–1^ with increasing O_2_ plasma time to 90 s and then increases to ∼6.4 × 10^–8^ m s^–1^ at 120 s and ∼1.3 × 10^–7^ m s^–1^ at 150 s. The PCTE+G+OP90+PDS and PCTE+G+OP120+PDS membranes show the highest permeance of KCl (∼5.1 × 10^–5^–6 × 10^–5^ m s^–1^) and B12 (∼2.8 × 10^–6^–4 × 10^–6^ m s^–1^) as well as low permeance of Lz (∼4 × 10^–8^–6.4 × 10^–8^ m s^–1^, Figure 2C), resulting in the highest selectivity for B12/Lz (up to ∼70) and KCl/Lz (up to ∼1280, Figure 2D).

The performance of the PCTE+G+OP90+PDS membrane was further evaluated using diffusive transport (see Methods) of a model dialysis biomolecule FITC-Ficoll 70 (abbreviated as Ficoll, molecular diameter ∼1.6–24 nm, Figure 3A) for up to 35 days of continuous operation, due to its more compact structure in comparison with other filtration probes like dextran.^55,71^ The permeates show Ficoll signals within 8 nm, indicating that the graphene membrane allows the transport of smaller Ficoll molecules (<8 nm) while rejecting larger Ficoll molecules (>8 nm, Figure 3A). The ratio of permeate over initial feed of Ficoll concentration was also computed in Figure 3B. For a nonselective membrane, the ratio would be a horizontal line across 0.5 in Y axis; i.e., the highest value that the ratio will reach is 0.5 for diffusion-driven transport. The permeate/initial feed ratio of the graphene membrane decreases significantly with the increase of Ficoll size (Figure 3B) and reaches near zero in the range >6–8 nm, indicating the size-dependent selectivity of Ficoll through the graphene membrane as well as excellent stability for the entire testing duration up to 35 days.

**Figure 3:**
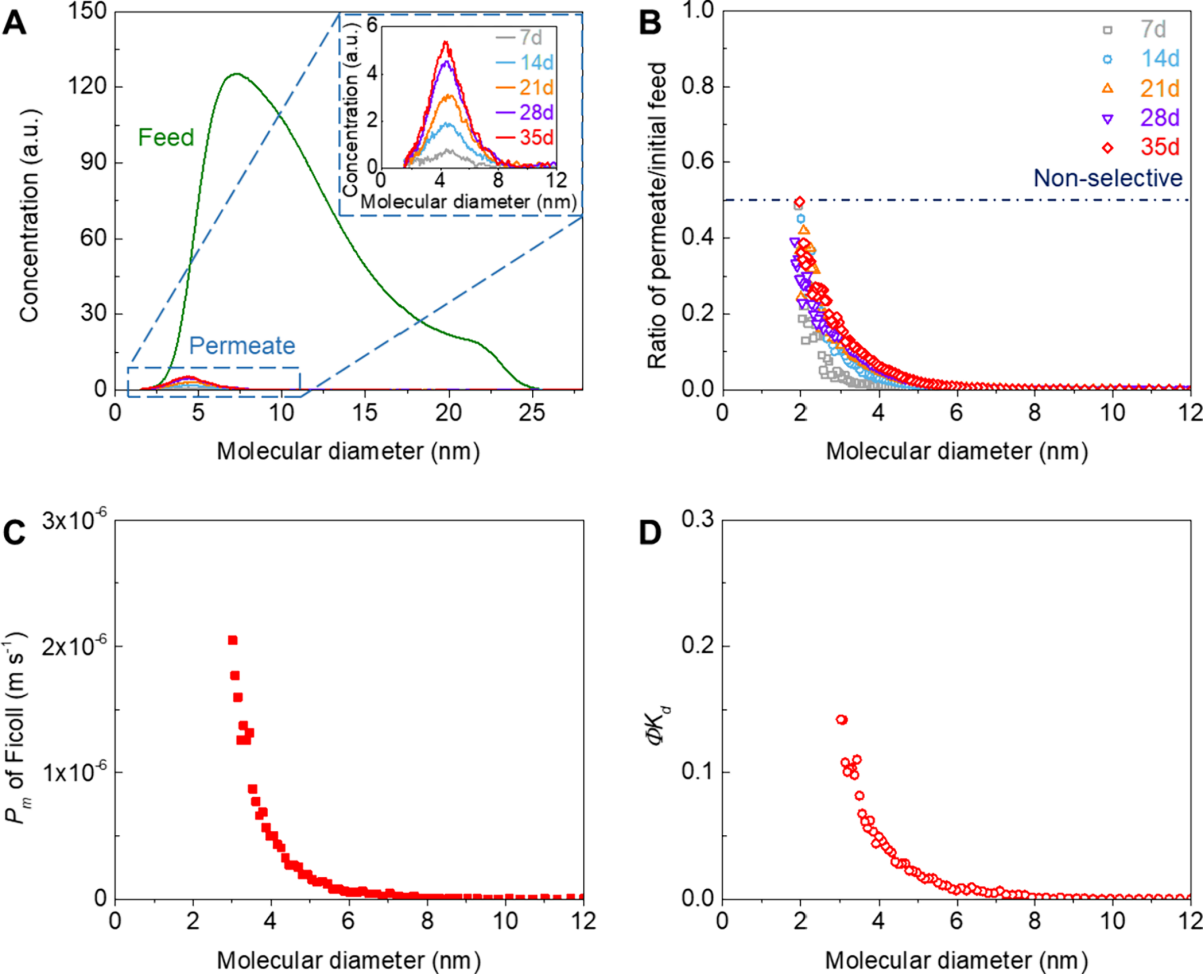
Ficoll transport through graphene NATMs. A) Ficoll concentrations in the feed and permeate (7, 14, 21, 28, and 35 days) solutions for PCTE+G+OP90+PDS membrane. B) Ratio of permeate/initial feed of Ficoll as a function of molecular diameter for PCTE+G+OP90+PDS membrane. C) Diffusive permeability (*P*_m_) and D) hindered diffusivity (Φ*K*_d_) as a function of molecular diameter for PCTE+G+OP90+PDS membrane. We note that the effective area accounting for PCTE support porosity of ∼10% is used to compute the *P*_m_ of PCTE+G+OP90+PDS membrane. Also, see Supporting Information, Figure S5.

The diffusive permeability (*P*_m_) and hindered diffusivity (*ΦK*_d_) of Ficoll were also computed by  and , respectively,^72,73^ while considering the PCTE support porosity ∼10% (see Methods and Figure 3C,D, also see Figure S5 without accounting for PCTE support porosity). The diffusive permeability for graphene membrane decreases with increasing solute molecular diameter. We note that PCTE+G+OP90+PDS membrane blocks most Ficoll molecules with molecular diameters >6–8 nm (Figure 3C), while almost completely rejecting Lz ∼3.8–4 nm (Figures 2B and 4A). Our observations indicate that, although more compact than dextran,^55,71^ Ficoll molecules are still more flexible/deformable than protein molecules^54,55^ such as Lz, thereby shifting the permeate curve toward larger molecular size.

**Figure 4:**
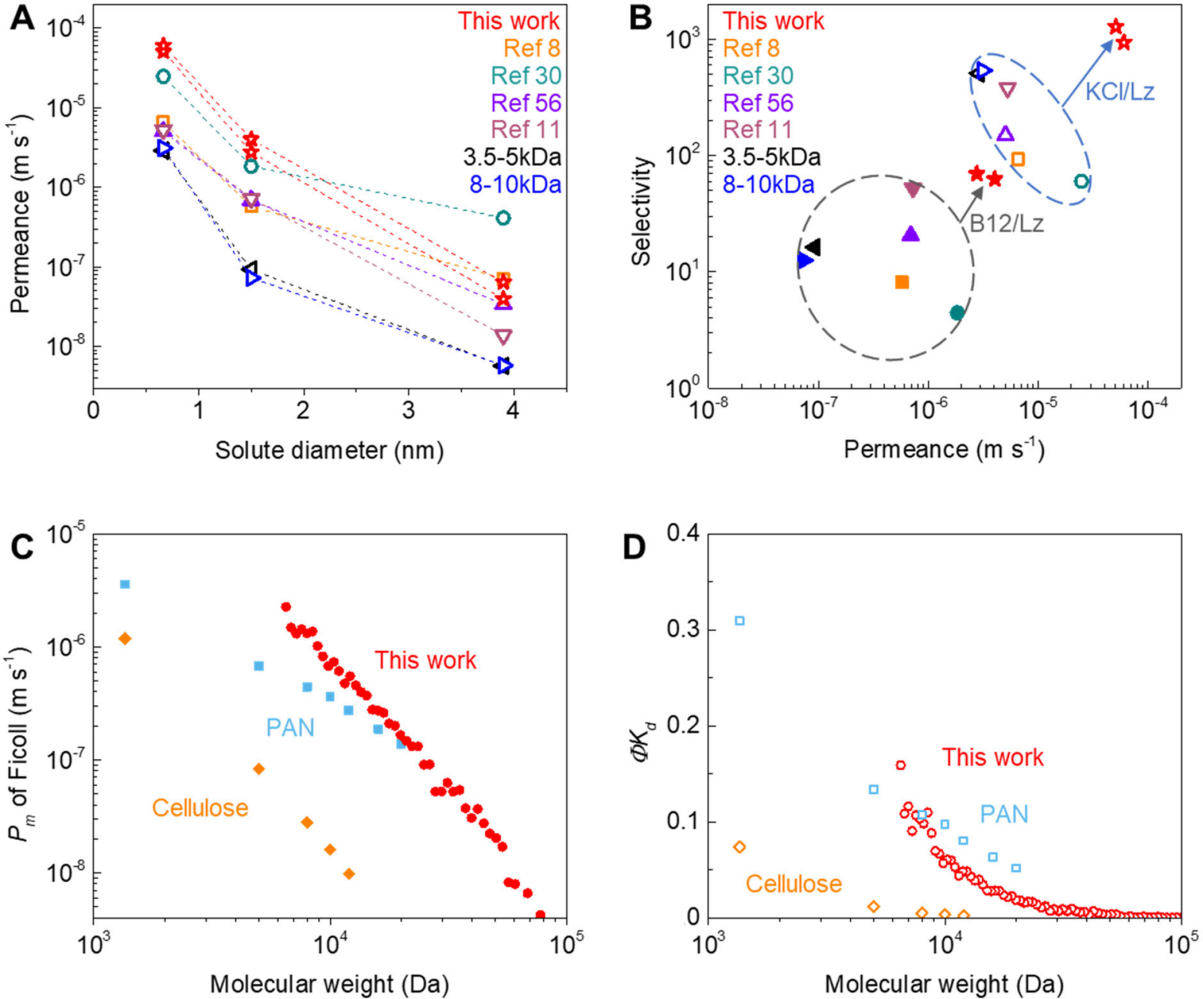
Performance evaluation and comparison of graphene membranes. A) Diffusive permeance (accounting for PCTE porosity of ∼10%) as a function of the solute diameter (KCl, hydrated diameter of K^+^ ∼0.662 and Cl^–^ ∼0.664 nm; B12, ∼1−1.5 nm; Lz ∼3.8–4 nm) using PCTE+G+OP90+PDS and PCTE+G+OP120+PDS membranes in this work compared with state-of-the-art commercially available dialysis membranes (3.5–5 kDa and 8–10 kDa)^11^ as well as laboratory-scale graphene membranes reported in the literature (PES+G,^11^ PES+G+IP+OP60,^56^ PAN-EP-G-IP-100,^8^ and PCTE+G+IP+OP45^30^). B) Diffusive permeance (accounting for PCTE support porosity of ∼10%) as a function of selectivity for B12/Lz (filled symbols, plotted with B12 permeance) and KCl/Lz (open symbols, plotted with KCl permeance) using PCTE+G+OP90+PDS and PCTE+G+OP120+PDS membranes in this work compared with state-of-the-art commercially available dialysis membranes (3.5–5 kDa and 8–10 kDa)^11^ as well as laboratory-scale graphene membranes reported in the literature (PES+G,^11^ PES+G+IP+OP60,^56^ PAN-EP-G-IP-100,^8^ and PCTE+G+IP+OP45^30^). Also, see Supporting Information, Figure S6. C) Diffusive permeability (*P*_m_, logarithmic scale) and D) hindered diffusivity (*ΦK*_d_) as a function of molecular weight for the PCTE+G+OP90+PDS membrane compared with conventional regenerated cellulose and PAN hemodialysis membranes.^75^ We note that effective areas accounting for PCTE support porosity of ∼10% are used to compute the *P*_m_ in all the membranes, and dextran (instead of Ficoll) was used for probing the performance of regenerated cellulose and PAN membranes in lietrature.^75^ Also, see Supporting Information Figure S6.

The partition coefficient (Φ) is defined as the ratio of solution concentration in the membrane to that in the external solution and the diffusive hindrance factor (*K*_d_) is defined as *D*/*D*_∞_ (solution diffusion coefficient through membrane/free solution diffusion coefficient). Hence, *ΦK*_d_ = 1 corresponds to no steric or diffusive hindrance (high diffusivity), and *ΦK*_d_ = 0 represents higher diffusive resistance (low diffusivity).^74^ The relatively large *ΦK*_d_ in the range of Ficoll molecular diameters <6–8 nm indicates low resistance to small and middle molecules, while much smaller *ΦK*_d_ in the range of Ficoll molecular diameters >6–8 nm indicates high resistance to large molecules (Figure 3D).

Further, we compare the performances of our graphene NATMs with commercial membranes as well as other state-of-the-art reports in the literature (Figure 4). Here, we note that an effective area of ∼10% is used to compute the permeance of membranes with PCTE supports in our work and in prior literature reports.^30^ Our membranes (PCTE+G+OP90+PDS and PCTE+G+OP120+PDS) show more than an order of magnitude higher permeance for all species (Figure 4A) as well as higher selectivity for B12/Lz (up to ∼70) and for KCl/Lz (up to ∼1280), compared to the state-of-the-art commercially available dialysis membranes (molecular weight cutoff ∼3.5–5 and ∼8–10 kDa,^11^ between the molecular weights of B12 ∼1−1.4 kDa and Lz ∼14.3 kDa). Our NATMs show significantly higher permeance for KCl and B12 and comparable permeances of Lz (Figure 4A) when compared to previously reported graphene membranes, i.e., PES+G,^11^ PES+G+IP+OP60,^56^ and PAN-EP-G-IP-100,^8^ as well as comparable permeances of KCl and B12, but much lower permeance of Lz in comparison to PCTE+G+IP+OP45 membrane in literature.^30^ Not only do these observations evidence the higher selectivity of our NATMs for KCl/Lz and B12/Lz (Figure 4B), but also the selectivity vs permeance plots for B12/Lz (filled symbols, plotted with B12 permeance) and KCl/Lz (open symbols, plotted with KCl permeance) show that our NATMs offer significant advances over state-of-the-art commercially available dialysis membranes as well as other graphene membranes reported in the literature for potential applications in dialysis, protein desalting, and small molecule separations, among others.

Finally, we compare the diffusive permeability (*P*_m_) and hindered diffusivity (*ΦK*_d_) of the PCTE+G+OP90+PDS membrane with conventional hemodialysis membranes including regenerated cellulose and polyacrylonitrile (PAN) membranes,^75^ while noting that effective areas accounting for membrane porosities are used to compute the *P*_m_ for all the membranes, and dextran is typically used for probing the performance of regenerated cellulose and PAN membranes in the literature.^75^ Compared to the regenerated cellulose membranes in the literature, our NATMs show a similarly decreasing trend with increasing solute molecular weight but up to 2 orders of magnitude higher permeability. However, when compared with PAN membrane, our NATMs show a steeper decrease with significantly higher permeability of small molecular weight solutes (∼2.3 × 10^–6^ m s^–1^ at MW ∼6.5 kDa) and much lower permeability of large molecular weight solutes (∼4.4 × 10^–9^ m s^–1^ at MW ∼66 kDa, Figure 4C). Additionally, our NATMs show significantly larger *ΦK*_d_ (high hindered diffusivity) compared to the regenerated cellulose membrane in the range of MW <50 kDa and negligible *ΦK*_d_ (low hindered diffusivity) in the range of MW >50 kDa, suggesting lower resistance to small and middle molecules and high resistance to large molecules (Figure 4D). Compared to the PAN membrane, our NATMs show higher hindered diffusivity (larger *ΦK*_d_) in the range of MW <8 kDa and lower hindered diffusivity (smaller *ΦK*_d_) in the range of MW >8 kDa, indicating smaller molecular weight cutoff and better selectivity (Figure 4D). In summary, the comparison of *P*_m_ and *ΦK*_d_ as a function of solute molecular weight plots shows our NATMs offer significant advances over conventional hemodialysis membranes (regenerated cellulose and PAN) with high permeability (low resistance) for small and middle molecules as well as negligible permeability (high resistance) for large molecules (MW >50 kDa), indicating potential for dialysis applications.

## Supporting Information Available

Method section. Figures S1: Experimental setup. Figure S2: Calculated pore size distribution of graphene lattice after O_2_ plasma (90 s) treatment. Figure S3: Comparison of diffusive transport between two distinct PCTE+G+OP90+PDS membranes. Figure S4: Measured diffusive permeance of the PCTE+G+OP(s)+PDS membranes. Figure S5: Measured diffusive permeability (*P*_m_) and hindered diffusivity (*ΦK*_d_) as a function of molecular diameter for the PCTE+G+OP90+PDS membrane. Figure S6: Measured data comparison of graphene membranes with state-of-the-art commercially available dialysis membranes as well as laboratory-scale graphene membranes reported in the literature (PDF)
