# Supplementary material for: Protein-Enabled Size-Selective Defect-Sealing of Atomically Thin 2D Membranes for Dialysis and Nanoscale Separations
Source: Nano Lett. 2024 Dec 23;25(1):193–203. doi: 10.1021/acs.nanolett.4c04706 (PMC11719630; doi:10.1021/acs.nanolett.4c04706)
Supplement: Supplementary file 1 — nl4c04706_si_001.pdf [file nl4c04706_si_001.pdf]

Supporting Information

Protein-Enabled Size-Selective Defect-Sealing of Atomically Thin 2D Membranes for Dialysis and Nanoscale Separations

Peifu Cheng,[†] *Nicholas Ferrell,*[‡] *Saban M. Hus,*[◊] *Nicole K. Moehring,*[∥] *Matthew J. Coupin,*[§] *Jamie Warner,*[§] *An-Ping Li,*[◊] *William H. Fissell,*[⊥ Δ] *Piran R. Kidambi**[† ∥ Δ # §]

[†]Department of Chemical and Biomolecular Engineering, Vanderbilt University, Nashville, Tennessee 37212, United States.

[‡]Division of Nephrology, Department of Internal Medicine, The Ohio State University Wexner Medical Center, Columbus, Ohio 43210, United States.

[◊]Center for Nanophase Materials Sciences, Oak Ridge National Laboratory, Oak Ridge, Tennessee 37831, United States.

[∥]Interdisciplinary Materials Science Program, Vanderbilt University, Nashville, Tennessee 37212, United States.

[⊥]Department of Medicine and Division of Nephrology and Hypertension, Vanderbilt University Medical Center, Nashville, Tennessee 37232, United States.

[Δ]Vanderbilt Institute of Nanoscale Sciences and Engineering, Vanderbilt University, Nashville, Tennessee 37212, United States.

[§]Walker Department of Mechanical Engineering, University of Texas at Austin, Austin, Texas 78712-1591, United States.

*E-mail: piran.kidambi@vanderbilt.edu

The supporting Information includes:
Methods
Supporting Figures S1-S6

METHODS

Graphene growth. Graphene was synthesized on Cu foils at 1060 °C using low-pressure chemical vapor deposition (LPCVD) as reported in detail elsewhere.[1–4] Polycrystalline Cu foil (~99.9% purity, ~2×7 cm^2, 18 μm thick, JX Holding HA) was sonicated in diluted nitric acid (~20%) for ~4 min to remove surface oxides and contaminants, followed by rinsing in deionized (DI) water for 2 min and drying in air.[1–4] Next, the Cu foil was loaded into a 1-inch hot-walled tube furnace and annealed at 1060 °C for 60 min under 100 sccm H_2. The graphene was grown under 2 sccm CH_4 and 100 sccm H_2 for 60 min, followed by another 60 min of growth with 4 sccm CH_4 and 100 sccm H_2. Finally, the foil was quench-cooled to room temperature in the final growth atmosphere.

Graphene transfer onto PCTE and TEM grids. Graphene transfer onto PCTE supports (~200 nm cylindrical pores, ~10% porosity, 10 μm thick, free of PVP coating, hydrophobic, Sterlitech Inc.) was performed via isopropanol-assisted hot lamination as described elsewhere.[1] Graphene on the bottom side of Cu foil was removed by pre-etching the foil in 0.2 M of ammonium persulfate (APS) solution for 15 min, followed by rinsing the foil in DI water (two times, 10 min per time) and drying it in air.[1–6] Next, PCTE was placed against the graphene/Cu foil with graphene side facing up and sandwiched between two pieces of weighing paper to make a paper/PCTE/graphene/Cu/paper stack. A small volume (~50 μL) of isopropanol (IPA) solvent was added to the PCTE/graphene interface before hot lamination. The stack was laminated with Teflon protective layers at 135 °C using a TruLam TL-320E laminator. After peeling off the weighing paper, the Cu foil was fully etched by floating the PCTE/graphene/Cu stack on APS solution. Finally, the PCTE/graphene stack was floated on DI water to remove APS residue, followed by rinsing in ethanol and drying in air.

Graphene transfer to Holey Carbon on 300 Mesh Au grids (Electron Microscopy Sciences) was performed using the polymer free method. The TEM grid was placed on as-synthesized CVD graphene on Cu foil with the CVD graphene removed from the bottom side by pre-etching in APS.[2,5,7–12] A drop of isopropyl alcohol was added to the stack and allow to evaporate to promote adhesion between the TEM grid and the CVD graphene on Cu foil. The stack was floated on 0.2M APS to etch the Cu foil using procedures detailed above. Finally, the TEM grid with CVD graphene was floated on DI water, followed by rinsing in ethanol and drying in air.

Graphene transfer onto SiO_2/Si wafer for Raman spectroscopy. Graphene transfer onto SiO_2/Si wafers was performed using sacrificial polymer scaffold transfer method.[1–6] First, CVD graphene on Cu foil was pre-etched to remove graphene on the bottom side as described above.[1–6] Polymethyl methacrylate (PMMA) in anisole (2 wt%) was drop-casted onto the graphene side of the pre-etched Cu foil, followed by drying in air. The foil was subsequently etched in APS solution to remove Cu, and the obtained PMMA/graphene stack was floated on DI water for 10 min. Finally, the stack was transferred onto a SiO_2 (300 nm)/Si wafer, followed by baking in air (up to 90 °C), washing in acetone and cleaning in IPA.

O_2 plasma treatment. O_2 plasma etching[2] was used to etch nanopores in CVD graphene transferred to PCTE and TEM grids. The etch was performed in pulses (15 s plasma followed by 120 s pause) in a plasma cleaner (Harrick Plasma, PDC-001) using low RF power of 7 W under 0.5 Torr O_2 pressure.

Protein-enabled defect sealing (PDS). PDS was performed using bovine serum albumin (BSA) in aqueous phase and trimesoyl chloride (TMC) in organic phase. Initially, the graphene/PCTE stack was annealed at 105 °C for 12 hours. Interfacial reaction was carried out in a Franz cell

(PermeGear, Inc., 15 mm orifice) by filling the bottom cell with BSA (Sigma Aldrich, A7906) in PBS (30 g/L) as the aqueous phase pH ~7.4, the graphene membrane was placed graphene-side down onto the solution and clamped into place. Subsequently, TMC (Alfa Aesar, 4422-95-1) in hexane (5 g/L) was added on the top side of the membrane as the organic phase. After reacting for 60 min, the membrane was rinsed thoroughly with hexane and then with ethanol and dried in air.[1–6 13]

Characterization. SEM images of graphene on PCTE supports were recorded by using a Zeiss Merlin Scanning Electron Microscope with a Gemini II Column operated at 2 kV. Raman spectra were acquired using a Thermo Scientific DXR Confocal Raman spectrometer with a 532 nm laser source using 1mW laser power. STM images were obtained with an Omicron variable temperature scanning tunneling microscope (VT-STM) at room temperature in the Center for Nanophase Materials Sciences at Oak Ridge National Laboratory.[5,6] The samples were annealed under vacuum at 500 °C for 1 h before STM imaging.

ADF-STEM images were acquired using a JEOL ARM200F NEOARM equipped with a CEOS ASCOR corrector operated at 80 kV. A 40 micron aperature was used to produce an electron probe with a convergence semiangle of 27 milliradians. The samples were annealed in vacuum ~175-200°C for 12 hours to minimize contaminations prior to imaging. The pore size of each nanopore in STEM images was computed[14] by converting the manually-measured pore area (A) into an effective diameter via $d_{pore} = \sqrt{4A/\pi}$ (Figure 1H). We also calculated the pore size (Figure S1) by adding the carbon electron diameter (0.13 nm) and substracting carbon van der Waals diameter (0.34 nm).[5,10,15,16] The pore density was determined by dividing the total number of pores imaged by the total effective area (not covered by contaminants) of the acquired images.

Solute transport measurements. Pressure-driven ethanol transport and diffusion-driven solute transport measurements across the fabricated membrane were performed as previously reported in detail elsewhere.[1–6,10,11,17,18] A customized 7 mL Side-Bi-Side glass diffusion cell (5 mm orifice, PermeGear, Inc.) with a gastight syringe (250 μL, Hamilton 1725 Luer Tip) installed on the left cell (leak-free connection, sealed with epoxy) was used for transport measurements (Figure S1). The membrane was installed between two diffusion cells with the graphene side facing the feed side (left cell), followed by clamping the cells in the diffusion system. During the measurement, the liquids in both cells were stirred vigorously at 1500 rpm with magnetic Teflon coated stir bars to minimize concentration polarization.

For measuring pressure-driven ethanol transport,[1,4,12,18] ethanol was used to wash the system three times before measurement. Both cells were subsequently filled with ethanol and a height difference was used to generate hydrostatic pressure driven flow through the membrane. A digital camera was used to record the change of ethanol meniscus level along the syringe every 1 min. The ethanol permeance was computed by $p = (\Delta V/\Delta P)/(\Delta t \times A_{effective})$, where p is the ethanol permeance, ΔV is the ethanol volume change (decrease), ΔP is the hydrostatic pressure difference across the membrane, Δt is the time interval (1 min), and $A_{effective}$ is the effective membrane area. The normalized flux was calculated by dividing the ethanol permeance of graphene membrane by that of PCTE substrate.[1,4,12,17,18]

Next, the system was washed with DI water for 5 times to completely replace ethanol residue and wet the PCTE cylindrical pores. Three model solutes covering a range of sizes (0.66–4 nm) were specifically selected for the transport measurement: KCl (salt, hydrated diameter of K^+ ~0.662 and Cl^- ~0.664 nm),[15] Vitamin B12 (B12, vitamin, ~1.5 nm, 1355 Da),[19] and Lysozyme (Lz, protein, ~3.8–4 nm, 14.3 kDa).[2]

For measuring KCl (Fisher Chemical, 7447-40-7) transport, KCl solution (0.5 mol L^{-1} in DI water) was filled into the feed side and DI water was filled into the permeate side, with a conductivity meter probe (attached to a Mettler Toledo SevenCompact S230 conductivity benchtop meter) immersed in the permeate side to measure the conductivity every 15 s for 15 min.[1–6,10,11,17] For measuring B12 (Sigma-Aldrich, 68-19-9) or Lz (Bio-world, 12650-88-3) transport, the organic molecule solution (1 mmol L^{-1} in 0.5 mol L^{-1} KCl) was filled into the feed side and KCl solution (0.5 mol L^{-1}) was filled into the permeate side, with a fiber optic dip probe (attached to an Agilent Cary 60 UV-vis Spectrophotometer) immersed in the permeate side to record the absorbance spectra of organic molecules (B12 or Lz) in the range of 190 to 1100 nm every 15 s for 40 min.[1–6,10,11,17] Different UV-vis wavelengths were used for measuring the intensity changes of corresponding species: 710 nm for DI water (reference wavelength), 360 nm for B12, and 282 nm for Lz, respectively. [1–6,10,11,17]

The flow rate of each solute was computed via the slope of concentration change in the permeate side, while the normalized flux was calculated by dividing the slope of the fabricated membrane by that of the PCTE support membrane.[1–6,10,11,17] The solute permeance was calculated using $P = \frac{V \times \frac{dC}{dt}}{\Delta C \times A_{effective}}$, where V is the volume of solution (7 mL), dC/dt is the slope of concentration change in the permeate side, $A_{effective}$ is the effective area of membrane subjected to diffusion test (5 mm diameter orifice area, accounting for 10% of PCTE porosity), and ΔC is the solute concentration difference across the membrane. All the measurements were repeated in triplicates to obtain average values and standard deviations.[1–6,10,11,17] Solute/solute selectivity between different species was obtained by calculating the ratio of their solute permeances.[1–6,10,11,17]

Preparation of Ficoll solutions. Ficoll 70 was tagged with fluorescein isothiocyanate (FITC) as described previously.[20] Briefly, 1 g of Ficoll 70 (Sigma-Aldrich, F2878) was dissolved in 20 mL of dimethyl sulfoxide (DMSO, Fisher Scientific, D128-500), followed by adding 20 mg of sodium bicarbonate (Fisher Scientific S233-500) and 100 mg of FITC (Sigma-Aldrich, F7250).[20] Next, the solution was heated in a boiling water bath for 15 min and was then poured slowly into 200 mL ethanol (200 proof, Sigma-Aldrich, E7023) and allowed to precipitate overnight while protecting it from exposure to light.[20] The precipitate was pelleted by centrifugation and the ethanol was removed. The pellet was dissolved in Milli-Q water (HPLC grade, J. T. Baker 4218-03) at 37°C until completely dissolved. Finally, the labeled FITC-Ficoll was eluted on an equilibrated desalting column (Sephadex G-25 PD-10) to removed unbound FITC.

Diffusion-driven dialysis experiment using Ficoll. Ficoll (0.05 mg/mL) in PBS solution (1×, 1 tablet dissolved in 200 mL water, pH ~7.4, Research Products International) was used as a neutral filtration probe in diffusion-driven dialysis experiment. The 7 mL Side-Bi-Side glass diffusion cell (Figure S1) was used for Ficoll transport measurement with similar mounting, rinsing and stirring processes as mentioned above. The four ports were sealed with parafilm tapes to prevent the water evaporation and ensure minimal feed concentration changes over time. The Ficoll feed and permeate solutions (0.1 mL for each time) were collected correspondingly from the feed and permeate cells after performing the experiment for 7, 14, 21, 28 and 35 days. The concentrations of Ficoll in the feed and permeate were analyzed by a size-exclusion chromatography with an Ultrahydrogel 500 column and guard column (Waters, Milford, MA).[21] PBS (150 mmol NaCl, 50 mmol phosphate, 200 ppm NaN_3, pH 7.0) was employed as the mobile phase with a flow rate of 0.5 mL min^{-1}. Ficoll was analyzed by a fluorescence detector (model G1321A, Agilent Technologies) at excitation/emission (Ex./Em.) 495/520 nm. The relationship between molecular

weight and retention time for each time point individually was obtained by using multi-angle light scattering (DAWN TREOS; Wyatt Technology, Santa Barbara, CA) along with differential refractive index (model G1362A, Agilent Technologies), and calculated using Agilent ChemStation software.[21]

The change in permeate concentration over time (t) was described by

$$\frac{dC_B}{d_t} = -\frac{P_m A(C_B - C_A)}{V_B}$$

where C_A and C_B are the concentrations of feed and permeate, respectively, P_m is the diffusive permeability, A is the membrane area, t is the time, and V_B is the volume of the permeate cell.

Integrating the equation gives[21–23]

$$\ln\left[\frac{C_B(t) - C_A}{C_B(0) - C_A}\right] = -\frac{AP_m t}{V_B}$$

where $C_B(t)$ and $C_B(0)$ are the permeate concentrations at different times, and t is the time interval between the two collections. The diffusive permeability in this work was computed using the initial feed and permeates at 7 and 14 days.

The relation between diffusive permeability P_m and hindered diffusivity ΦK_d was determined by[21,22,24]

$$\Phi K_d = \frac{P_m \delta}{D_\infty}$$

where Φ is the partition coefficient, K_d is the diffusive hindrance factor, P_m is the diffusive permeability, δ is the membrane thickness, and D_∞ is the free solution diffusion coefficient, which was calculated by[21,24]

$$D_\infty = \frac{kT}{6\pi\eta r_s}$$

where k is the Boltzmann's constant, T is the absolute temperature (K), and η is the media viscosity (1×10^{-3} Pa·s) and r_s is the Stokes-Einstein radius.

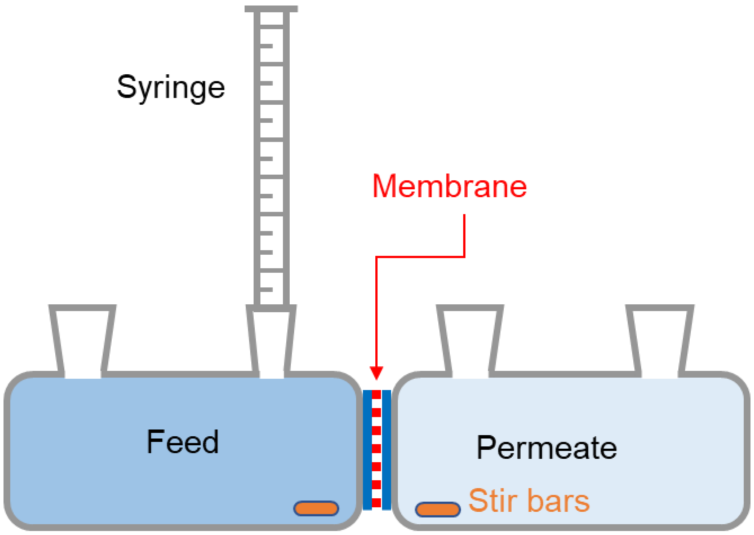

Figure S1. Experimental setup to measure diffusive transport.

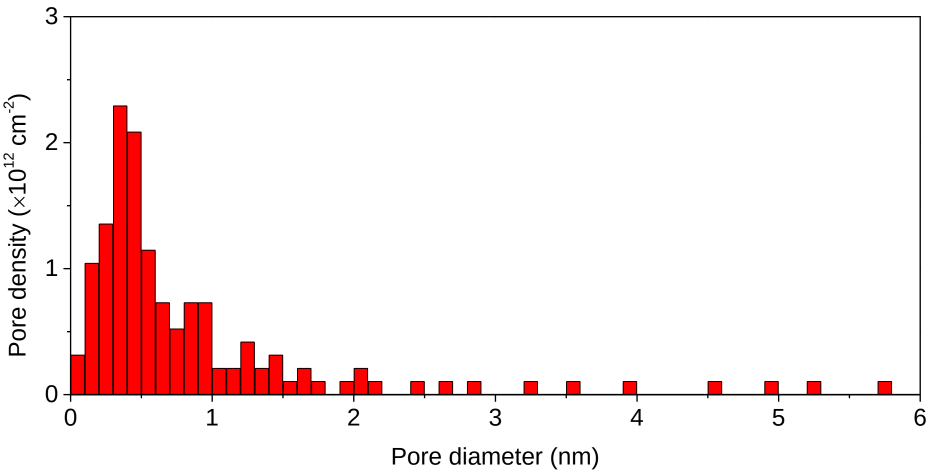

Figure S2. Calculated pore size distributions of graphene after O_2 plasma treatment for 90 s. The calculated pore diameter was obtained by adding carbon electron diameter (~0.13 nm) to the measured pore diameter and then subtracting carbon van der Waals diameter (~0.34 nm).[5,10,12] Also see Figure 1H for measured pore size distribution.

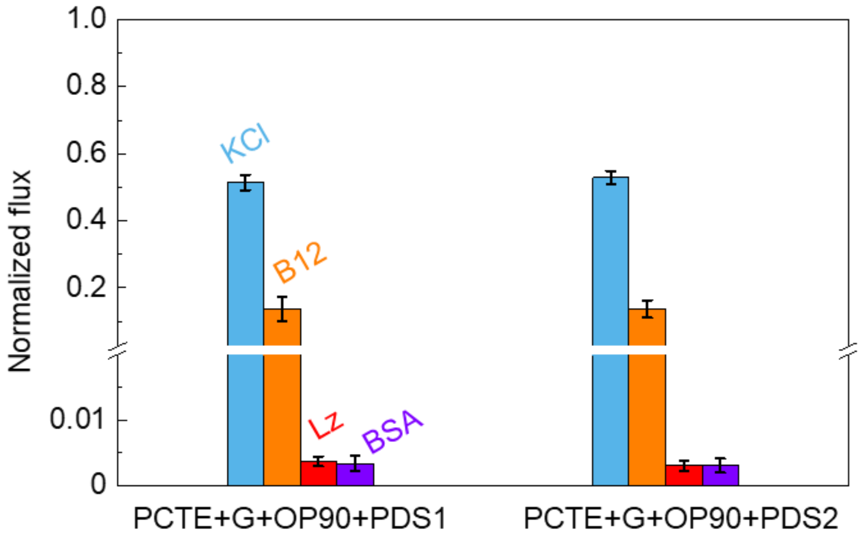

Figure S3. Comparison of diffusive transport between two distinct PCTE+G+OP90+PDS membranes. These results show that the normalized fluxes of different NATMs fabricated by the same protocol are consistent, indicating the reliability and reproducibility of the entire process comprising graphene synthesis, graphene transfer, O_2 plasma treatment and PDS.

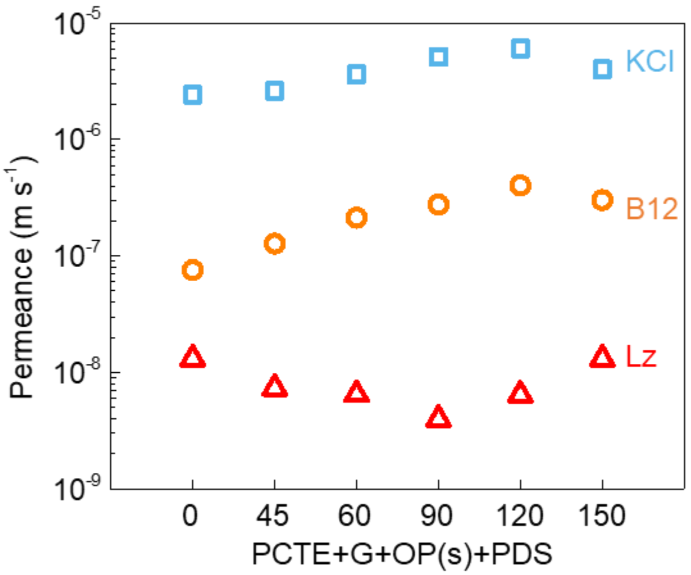

Figure S4. Diffusive permeance (without accounting for PCTE support porosity of ~10%) of the PCTE+G+OP(s)+PDS membranes.

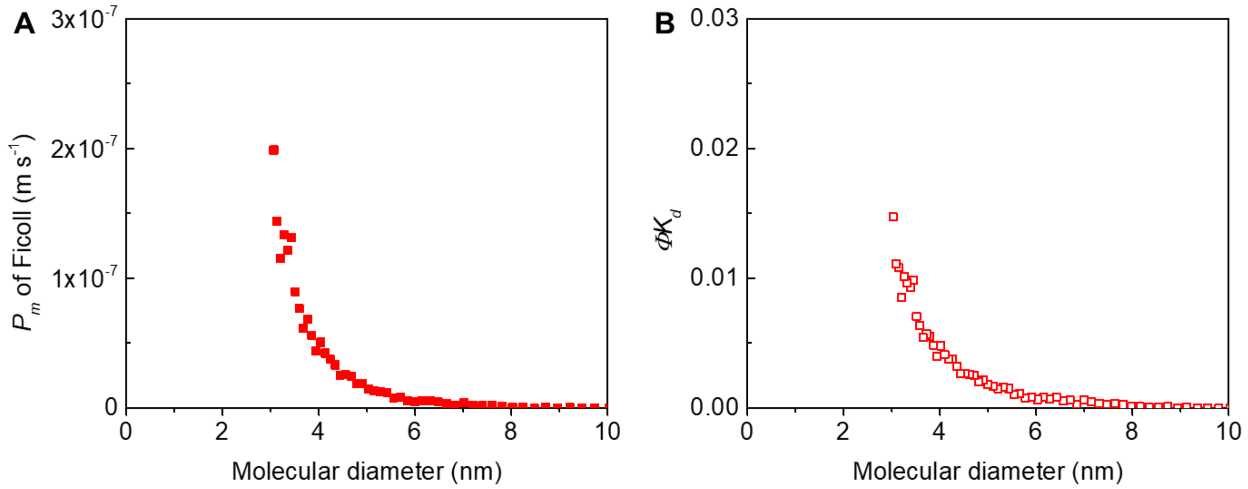

Figure S5. A) Diffusive permeability (P_m) and B) hindered diffusivity (ΦK_d) as a function of molecular diameter for the PCTE+G+OP90+PDS membrane as measured (without accounting for PCTE support porosity of ~10%, also see Figure 3).

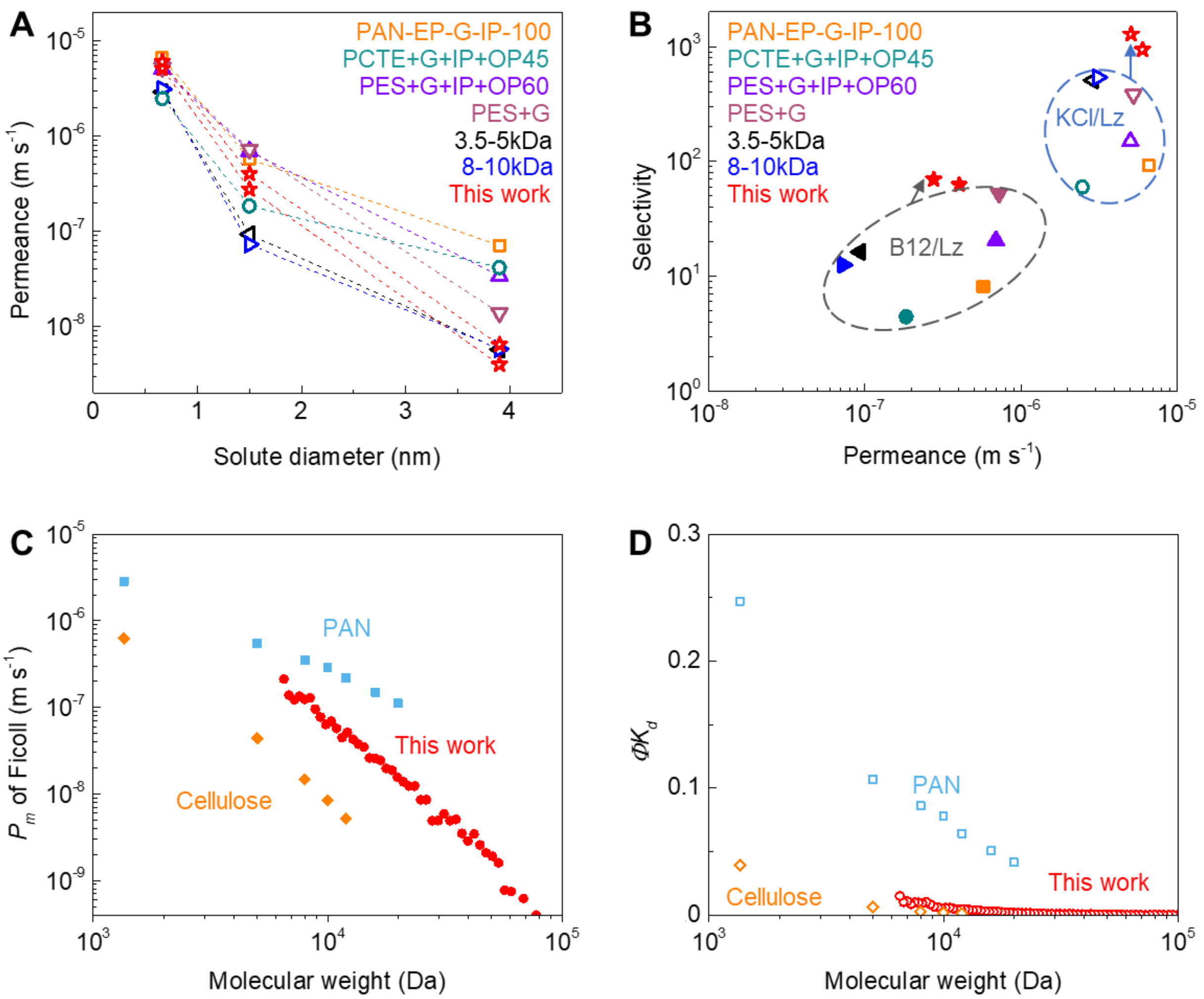

Figure S6. A) Diffusive permeance (without accounting for PCTE support porosity of ~10%) as a function of the solute diameter (KCl, hydrated diameter of K^+ ~0.66 nm and Cl^- ~0.66 nm; B12, ~1.5 nm; Lz ~3.8-4 nm) using PCTE+G+OP90+PDS and PCTE+G+OP120+PDS membranes in this work compared with state-of-the-art commercially available dialysis membranes (3.5-5 kDa and 8-10 kDa)[6] as well as other graphene membranes reported in the literature (PES+G,[6] PES+G+IP+OP60,[3] PAN-EP-G-IP-100,[25] and PCTE+G+IP+OP45[1]). B) Selectivity as a function of diffusive permeance (without accounting for PCTE support porosity of ~10%). Selectivity of B12/Lz (filled symbols plotted with B12 permeance) and KCl/Lz (open symbols plotted with KCl permeance) using PCTE+G+OP90+PDS and PCTE+G+OP120+PDS membranes in this work compared with state-of-the-art commercially available dialysis membranes (3.5-5 kDa and 8-10 kDa)[6] as well as other graphene membranes reported in the literature (PES+G,[6] PES+G+IP+OP60,[3] PAN-EP-G-IP-100,[25] and PCTE+G+IP+OP45[1]). C) Diffusive permeability

(P_m) and D) hindered diffusivity (ΦK_d) without accounting for PCTE support porosity of ~10% as a function of molecular weight for PCTE+G+OP90+PDS membrane compared with conventional cellulose and PAN hemodialysis membranes.[26] Note dextran (instead of Ficoll) was used for probing the performance of cellulose and PAN membranes in literature.[26] Also, see Figure 4.

References

(1) Cheng, P.; Moehring, N. K.; Idrobo, J. C.; Ivanov, I. N.; Kidambi, P. R. Scalable Synthesis of Nanoporous Atomically Thin Graphene Membranes for Dialysis and Molecular Separations via Facile Isopropanol-Assisted Hot Lamination. *Nanoscale* **2021**, *13* (5), 2825–2837.

(2) Kidambi, P. R.; Jang, D.; Idrobo, J.-C.; Boutilier, M. S. H.; Wang, L.; Kong, J.; Karnik, R. Nanoporous Atomically Thin Graphene Membranes for Desalting and Dialysis Applications. *Adv. Mater.* **2017**, *29* (33), 1700277.

(3) Kidambi, P. R.; Mariappan, D. D.; Dee, N. T.; Vyatskikh, A.; Zhang, S.; Karnik, R.; Hart, A. J. A Scalable Route to Nanoporous Large-Area Atomically Thin Graphene Membranes by Roll-to-Roll Chemical Vapor Deposition and Polymer Support Casting. *ACS Appl. Mater. Interfaces* **2018**, *10* (12), 10369–10378.

(4) Kidambi, P. R.; Terry, R. A.; Wang, L.; Boutilier, M. S. H.; Jang, D.; Kong, J.; Karnik, R. Assessment and Control of the Impermeability of Graphene for Atomically Thin Membranes and Barriers. *Nanoscale* **2017**, *9* (24), 8496–8507.

(5) Cheng, P.; Kelly, M. M.; Moehring, N. K.; Ko, W.; Li, A.-P.; Idrobo, J. C.; Boutilier, M. S. H.; Kidambi, P. R. Facile Size-Selective Defect Sealing in Large-Area Atomically Thin Graphene Membranes for Sub-Nanometer Scale Separations. *Nano Lett.* **2020**, *20* (8), 5951–5959.

(6) Kidambi, P. R.; Nguyen, G. D.; Zhang, S.; Chen, Q.; Kong, J.; Warner, J.; Li, A.-P.; Karnik, R. Facile Fabrication of Large-Area Atomically Thin Membranes by Direct Synthesis of Graphene with Nanoscale Porosity. *Adv. Mater.* **2018**, *30* (49), 1804977.

(7) Hauwiller, M. R.; Ondry, J. C.; Alivisatos, A. P. Using Graphene Liquid Cell Transmission Electron Microscopy to Study in Situ Nanocrystal Etching. *JoVE* **2018**, No. 135, e57665.

(8) Park, J.; Elmlund, H.; Ercius, P.; Yuk, J. M.; Limmer, D. T.; Chen, Q.; Kim, K.; Han, S. H.; Weitz, D. A.; Zettl, A.; Alivisatos, A. P. 3D Structure of Individual Nanocrystals in Solution by Electron Microscopy. *Science* **2015**, *349* (6245), 290.

(9) Regan, W.; Alem, N.; Alemán, B.; Geng, B.; Girit, Ç.; Maserati, L.; Wang, F.; Crommie, M.; Zettl, A. A Direct Transfer of Layer-Area Graphene. *Appl. Phys. Lett.* **2010**, *96* (11), 113102.

(10) O'Hern, S. C.; Jang, D.; Bose, S.; Idrobo, J.-C.; Song, Y.; Laoui, T.; Kong, J.; Karnik, R. Nanofiltration across Defect-Sealed Nanoporous Monolayer Graphene. *Nano Lett.* **2015**, *15* (5), 3254–3260.

(11) O'Hern, S. C.; Boutilier, M. S. H.; Idrobo, J.-C.; Song, Y.; Kong, J.; Laoui, T.; Atieh, M.; Karnik, R. Selective Ionic Transport through Tunable Subnanometer Pores in Single-Layer Graphene Membranes. *Nano Lett.* **2014**, *14* (3), 1234–1241.

(12) Cheng, P.; Fornasiero, F.; Jue, M. L.; Ko, W.; Li, A.-P.; Idrobo, J. C.; Boutilier, M. S. H.; Kidambi, P. R. Differences in Water and Vapor Transport through Angstrom-Scale Pores in Atomically Thin Membranes. *Nat. Commun.* **2022**, *13* (1), 6709.

(13) Zhao, J.; Zhang, Y.; Su, Y.; Liu, J.; Zhao, X.; Peng, J.; Jiang, Z. Cross-Linked Bovine Serum Albumin Composite Membranes Prepared by Interfacial Polymerization with Stimuli-Response Properties. *J. Memb. Sci.* **2013**, *445*, 1–7.

(14) Cohen-Tanugi, D.; Grossman, J. C. Water Desalination across Nanoporous Graphene. *Nano Lett.* **2012**, *12* (7), 3602–3608.

(15) Wang, L.; Boutilier, M. S. H.; Kidambi, P. R.; Jang, D.; Hadjiconstantinou, N. G.; Karnik, R. Fundamental Transport Mechanisms, Fabrication and Potential Applications of

Nanoporous Atomically Thin Membranes. *Nat. Nanotechnol.* **2017**, *12* (6), 509–522.
(16) Jang, D.; Idrobo, J.-C.; Laoui, T.; Karnik, R. Water and Solute Transport Governed by Tunable Pore Size Distributions in Nanoporous Graphene Membranes. *ACS Nano* **2017**, *11* (10), 10042–10052.
(17) O'Hern, S. C.; Stewart, C. A.; Boutilier, M. S. H.; Idrobo, J.-C.; Bhaviripudi, S.; Das, S. K.; Kong, J.; Laoui, T.; Atieh, M.; Karnik, R. Selective Molecular Transport through Intrinsic Defects in a Single Layer of CVD Graphene. *ACS Nano* **2012**, *6* (11), 10130–10138.
(18) Kidambi, P. R.; Boutilier, M. S. H.; Wang, L.; Jang, D.; Kim, J.; Karnik, R. Selective Nanoscale Mass Transport across Atomically Thin Single Crystalline Graphene Membranes. *Adv. Mater.* **2017**, *29* (19), 1605896.
(19) Ferris, C. J.; Panhuis, M. in het. Diffusion of Vitamin B12 in Gellan Gum-Carbon Nanotube Hydrogels. In *2010 International Conference on Nanoscience and Nanotechnology*; 2010; pp 234–236.
(20) Ohlson, M.; Sörensson, J.; Haraldsson, B. Glomerular Size and Charge Selectivity in the Rat as Revealed by FITC-Ficoll and Albumin. *Am. J. Physiol. Physiol.* **2000**, *279* (1), F84–F91.
(21) Ferrell, N.; Cameron, K. O.; Groszek, J. J.; Hofmann, C. L.; Li, L.; Smith, R. A.; Bian, A.; Shintani, A.; Zydney, A. L.; Fissell, W. H. Effects of Pressure and Electrical Charge on Macromolecular Transport Across Bovine Lens Basement Membrane. *Biophys. J.* **2013**, *104* (7), 1476–1484.
(22) Edwards, A.; Deen, W. M.; Daniels, B. S. Hindered Transport of Macromolecules in Isolated Glomeruli. I. Diffusion across Intact and Cell-Free Capillaries. *Biophys. J.* **1997**, *72* (1), 204–213.
(23) Robertson, B. C.; Zydney, A. L. Hindered Protein Diffusion in Asymmetric Ultrafiltration Membranes with Highly Constricted Pores. *J. Memb. Sci.* **1990**, *49* (3), 287–303.
(24) Wang, D.; Sant, S.; Ferrell, N. A Biomimetic In Vitro Model of the Kidney Filtration Barrier Using Tissue-Derived Glomerular Basement Membrane. *Adv. Healthc. Mater.* **2021**, *10* (16), 2002275.
(25) Shen, L.; Shi, Q.; Zhang, S.; Gao, J.; Cheng, D. C.; Yi, M.; Song, R.; Wang, L.; Jiang, J.; Karnik, R.; Zhang, S. Highly Porous Nanofiber-Supported Monolayer Graphene Membranes for Ultrafast Organic Solvent Nanofiltration. *Sci. Adv.* **2022**, *7* (37), eabg6263.
(26) Langsdorf, L. J.; Zydney, A. L. Diffusive and Convective Solute Transport through Hemodialysis Membranes: A Hydrodynamic Analysis. *J. Biomed. Mater. Res.* **1994**, *28* (5), 573–582.
